# Microbial product translocation and mortality in adults hospitalised with HIV-associated tuberculosis: a prospective observational cohort study

**DOI:** 10.21203/rs.3.rs-9856875/v1

**Published:** 2026-06-01

**Authors:** Charlotte Schutz, Artur T. L. Queiroz, Tiago F. Mota, Amy Ward, David Barr, Saskia Janssen, Muki Shey, Robert J. Wilkinson, Katalin Wilkinson, Rosie Burton, Benjamin Lelouvier, Bruno B. Andrade, Graeme Meintjes

**Affiliations:** 1 Wellcome Centre for Infectious Diseases Research in Africa, Institute of Infectious Disease and Molecular Medicine, University of, Cape Town, Observatory, South Africa; 2 Department of Medicine, University of Cape Town, Observatory, South Africa; 3 Multinational Organization Network Sponsoring Translational and Epidemiological Research (MONSTER) Institute, Salvador, Brazil; 4 Laboratório de Pesquisa Clínica e Translacional, Instituto Gonçalo Moniz, Fundação Oswaldo Cruz, Salvador, Brazil; 5 Freie Universität Berlin, Institute for Parasitology and Tropical Veterinary Medicine, Berlin, Germany; 6 Freie Universität Berlin, Veterinary Centre for Resistance Research, Berlin, Germany; 7 Wellcome Trust Liverpool Glasgow Centre for Global Health Research, University of Liverpool, Liverpool, United Kingdom; 8 Amsterdam University Medical Center, University of Amsterdam, Amsterdam, the Netherlands; 9 The Francis Crick Institute, London, United Kingdom; 10 Department of Medicine, Imperial College, London, United Kingdom; 11 AMR Learning Initiatives, MSF Academy, Cape Town, South Africa; 12 Vaiomer, Labege, France; 13 Division of Infectious Diseases, Department of Medicine, Johns Hopkins University, Baltimore, Maryland, United States of America; 14 Department of International Health, Bloomberg School of Public Health, Johns Hopkins University, Baltimore, Maryland, United States of America; 15 Blizard Institute, Faculty of Medicine and Dentistry, Queen Mary University of London, London, UK

**Keywords:** HIV-associated tuberculosis, microbial translocation, blood microbiome, metagenomics

## Abstract

**Background:**

HIV-associated tuberculosis (HIV-TB) results in unacceptably high mortality rates despite appropriate treatment. Patients hospitalized with HIV-TB often have disseminated tuberculosis and sepsis syndrome which may result in gastro-intestinal barrier dysfunction and facilitate microbial product translocation. Microbial product translocation may contribute to HIV-TB deaths by driving systemic inflammation.

**Objectives:**

To assess microbial product translocation and gastrointestinal epithelial damage in patients hospitalized with HIV-TB and the association with 12-week mortality and biomarkers of tuberculosis dissemination. To describe the bacterial blood microbiome (abundance and diversity) in patients with HIV-TB, its association with mortality and tuberculosis dissemination and compare to outpatient controls.

**Methods:**

Patients hospitalized with a new diagnosis of HIV-TB were enrolled and prospectively followed for 12 weeks. Markers of microbial product translocation and gastrointestinal damage were measured in a subset (n=373) and bacterial 16s rDNA was quantitated and metagenomic sequencing performed in 235 patients. Microbial product translocation and gastrointestinal epithelial damage marker concentrations were compared between hospitalized patients who died and survivors and inpatients compared to HIV-positive outpatient controls. Logistic regression analysis was performed to determine associations with mortality. Bacterial abundance, diversity and immune perturbation was measured and analysed across patient outcome groups and in patients with tuberculosis dissemination.

**Results:**

Patients hospitalized with HIV-TB had significantly higher concentrations of bacterial 16s rDNA, soluble CD14 (sCD14), lipopolysaccharide binding protein (LBP), trefoil factor 3 (TFF3) and lower endotoxin core antibody IgM (EndoCAB), compared to outpatient controls. Soluble CD14 and TFF3 were significantly higher and EndoCAB lower in inpatients who died versus survivors. TFF3 was independently associated with mortality. LPS, sCD14, LBP, EndoCAB and TFF3 showed significant trends in patients with positive biomarkers of tuberculosis dissemination. Metagenomic sequencing showed higher diversity in hospitalised HIV-TB patients compared to controls, but diversity was not different between outcome groups. *Mycobacterium* genus proportions were increased in hospitalised patients who died compared to survivors.

**Conclusion:**

We found evidence of increased gastrointestinal epithelial damage and microbial product translocation in patients hospitalized with HIV-TB and in patients with positive biomarkers for tuberculosis dissemination, however, only TTF3 (a marker of gastrointestinal epithelial damage), was independently associated with mortality.

## Background:

Tuberculosis is the leading cause of hospitalization and death in HIV-positive people worldwide. HIV-associated tuberculosis (HIV-TB) was responsible for 12% of global TB mortality in 2024 and 72% of global HIV-TB deaths occurred in the Africa Region^1–3^. Mechanisms underlying mortality are incompletely understood, although disseminated *Mycobacterium tuberculosis* load and the host immune response have been associated with early mortality^4^.

During acute HIV infection, there is early and profound damage to the structural and immunological barriers of the gastrointestinal tract that persists during chronic HIV^5,6^. HIV-associated damage allows increased gastrointestinal microbial product translocation into peripheral blood and is associated with a distinct blood microbial signature^7^. Microbial product translocation contributes to chronic systemic immune activation, auto-antibody production and is associated with disease progression and mortality^5,6,8–11^. The composition of translocated microbiota and the resultant inflammatory response impacts CD4 T-cell recovery on antiretroviral therapy^12–14^.

Translocation of bacterial products (such as lipopolysaccharide and flagellin) is not generally associated with bacteremia (positive bacterial blood cultures), but has been shown to drive systemic inflammation in conditions such as inflammatory bowel disease, visceral leishmaniasis, graft versus host disease, extensive burn wounds, hemorrhagic shock and acute alcohol intoxication^6,15–20^. In Crohn’s disease and liver cirrhosis the detection of bacterial DNA in blood is associated with adverse clinical endpoints such as relapse and mortality respectively^21,22^. Patients with severe HIV-TB may have additional damage to gastrointestinal mucosal integrity due to sepsis syndrome^23–25^, resulting in intestinal hypoperfusion. Additionally, intestinal tuberculosis is present in 36–43% of post-mortem HIV-TB cases^26,27^ and can affect the mucosa and sub-mucosal layers. It is plausible that increased microbial product translocation may be the harbinger of the translocation of whole viable bacteria from the gastrointestinal tract into the bloodstream resulting in deaths.

Microbial product translocation can be measured directly by quantifying microbial products such as LPS or bacterial 16s ribosomal DNA (16s rDNA) in peripheral blood^7,28,29^. Indirect measures of microbial translocation infer the degree of microbial translocation by measuring concentrations of soluble mediators, binding proteins and antibodies which reflect the immune response to LPS, such as soluble CD14 (sCD14), lipopolysaccharide binding protein (LBP) and endotoxin core antibody (EndoCAB).^6,28,30,31^ Biomarkers reflecting damage to the gastrointestinal mucosal lining have also been used to infer the degree of microbial translocation. During acute HIV infection microbial translocation is limited with low concentrations of LPS and raised sCD14, LBP and EndoCAB concentrations, whereas EndoCAB concentrations decrease and LPS concentrations increase during chronic HIV infection^32^.

Lipopolysaccharide (LPS or endotoxin) is an integral part of the gram-negative bacterial cell wall and stimulates monocyte/macrophages by binding to cell-surface CD14 receptors. CD14 binds LPS to the toll-like receptor 4 (TLR4)/MD2 complex which triggers a cascade that results in the production of inflammatory cytokines. An exaggerated immune response to LPS may be harmful, thus there are also efficient mechanisms to clear LPS from the circulation while limiting the immune response^33^. Bacterial 16s ribosomal DNA (16s rDNA), part of the chromosomal DNA, is a conserved DNA sequence in bacterial species which encodes 16s ribosomal RNA. It is essential for bacterial survival and function and is used to identify and classify bacteria^34^. Soluble CD14 (sCD14) is produced by activated monocytes and macrophages and either facilitates a pro-inflammatory immune response via membrane bound CD14 and the TLR4/MD2 complex or facilitates clearance of LPS from blood thereby limiting the immune response^35^. Higher concentration of sCD14 is a non-specific marker of monocyte and macrophage activation and is regarded as an indirect marker of the presence of LPS immune stimulation. Lipopolysaccharide binding protein (LBP) is an acute phase protein which, similar to sCD14, can facilitate an immune response to LPS or clear it from the circulation without stimulating an immune response^36^. Endotoxin core antibody (EndoCAB) is an antibody which is involved in clearing LPS from systemic circulation. EndoCAB which is bound to LPS is not measurable by the EndoCAB assay, thus lower concentrations of EndoCAB are regarded as indicating higher concentrations of LPS in the circulation. TFF3 is a secretory protein which is produced by mucous secreting cells of the intestinal mucosa^37^ and concentrations increase with gastrointestinal mucosal damage. Intestinal fatty acid binding proteins (IFABP) are cytoplasmic proteins in intestinal epithelial cells and are rapidly released into systemic circulation when enterocyte necrosis occurs^38^.

In this study, we measured concentrations of two direct, three indirect markers of microbial translocation, two markers of gastrointestinal epithelial damage and performed metagenomic sequencing on bacterial DNA extracted from whole blood of patients hospitalized with HIV-TB and HIV-positive outpatient controls without tuberculosis. We determined 12-week mortality in hospitalized patients. We hypothesized that in patients hospitalized with severe HIV-TB these biomarkers would demonstrate more severe damage to the gastrointestinal epithelium and increased microbial product translocation, and that these differences would be associated with mortality. We also hypothesized that patients with disseminated tuberculosis (tuberculosis which has spread through the blood stream to multiple organs) would have values reflecting more severe damage to the gastrointestinal endothelium and microbial product translocation compared to patients without disseminated tuberculosis. We analysed blood bacterial DNA by measuring 16s rDNA concentrations and bacterial genus identification in patients hospitalized with HIV-TB and hypothesized that patients who died would have higher concentration of microbial products with higher diversity.

## Patients and methods

### Study design and study population

We enrolled non-pregnant, HIV-positive adults with CD4 count of ≤350 cells/μl admitted to Khayelitsha Hospital, South Africa with a new diagnosis of tuberculosis and ambulant HIV-positive outpatients without tuberculosis at Ubuntu clinic, Site B Khayelitsha, between January 2014 and October 2016. Hospitalized patients were recruited as part of an observational cohort study investigating causes of mortality in hospitalized patients with HIV-TB^39^: HIV-positive adults with a suspected new diagnosis of tuberculosis were enrolled and samples collected upon admission. Outpatient controls were enrolled and samples collected during a routine visit to the primary care antiretroviral clinic. Controls were screened to exclude tuberculosis using a symptom screen, sputum (if able to produce) and urine Xpert MTB/RIF testing. Hospitalized patients were followed up 4 weeks and at 12 weeks to ascertain survival status. Markers were measured on stored samples in a randomly selected subset of hospitalized participants with HIV-TB and in HIV-positive outpatient controls without tuberculosis. Bacterial 16s rDNA concentrations were measured and metagenomic sequencing performed in a smaller cohort which included all hospitalized patients in the selected subset who died and had a stored whole blood sample plus a random selection of hospitalized survivors and the outpatient controls.

### Laboratory methods

All samples were collected at baseline upon enrolment to the study. Blood tests were performed by the National Health Laboratory Service (NHLS) as previously reported^39^. Concentrated urine samples were tested using the Xpert MTB/RIF assay. Mycobacterial blood cultures were performed using Myco/F Lytic bottles (Becton Dickinson Biosciences). The Genotype MTBDRplus assay (Hain Lifesciences) was used to identify *Mycobacterium tuberculosis* complex from positive blood cultures. Urine lipoarabinomannan (LAM) testing was performed by an independent laboratory on stored urine samples using Alere Determine TB LAM antigen test. The test strips were read by two independent readers who were blinded to clinical details and outcome. After the initial independent reading, results were compared and in case of discordant results, the test strip was reviewed to establish a mutually agreed consensus result. Peripheral blood samples were collected in tubes containing ethylenediaminetetraacetic acid (EDTA) and centrifuged at 3000 rpm for 5 minutes to derive plasma that was stored at −80°C. Whole blood samples were also stored at −80°C.

The Limulus Amebocyte Lysate (LAL) QCL-1000 (Lonza) assay was used to measure LPS concentration^40^. An optimized protocol using pyrogen free consumables was used. sCD14 was measured using the R&D Quantikine ELISA at a 1:1000 sample dilution. LBP and EndoCAB IgM were measured using the Hycult Biotech ELISA kit at 1:10000 and 1:100 sample dilutions, respectively, following manufacturer’s instructions. TFF3 and IFABP were measured using R&D Quantikine ELISA kits at a 1:50 sample dilution and the Hycult Biotech ELISA with a 1:2 sample dilution, respectively. Sample dilution experiments were performed to determine the optimal dilution when appropriate. In the ELISA assays concentrations which were higher than the range of the standard curve were imputed as the highest value of the standard curve and concentrations below the range of the standard curve were imputed as half of the lowest value of the standard curve. Soluble inflammatory mediators were measured on the Biorad Bioplex 200 Luminex platform using the Bio-Plex Pro Human Cytokine Standard 27-Plex kit (Group I) using a 1:2 dilution as previously reported^4^.

DNA from whole blood was isolated and amplified in a strictly controlled environment at Vaiomer SAS (Labège, France) using a stringent contamination-aware approach as described previously^41–43^. Real-time quantitative polymerase chain reaction (qPCR) amplification was performed using 16S universal primers targeting the hypervariable V3-V4 region of the bacterial 16S ribosomal gene as described previously.^41,43^

The microbial populations based on rDNA present in blood were determined using next-generation sequencing of V3-V4 variable regions of the 16S rRNA bacterial gene as previously described.^41,44^ For each sample, a sequencing library was generated by addition of sequencing adapters. The joint pair length was set to encompass a 467 base pairs amplicon (using Escherichia coli 16S as a reference) with a 2 × 300 paired end MiSeq kit V3 (Illumina, San Diego, CA, USA). The detection of the sequencing fragments was performed using the MiSeq Illumina^®^ technology.

The raw sequencing data was filtered by short reads, adapters, quality scoring and ambiguous base calls were removed using Trimmomatic^45^ software and the FastX-toolkit (http://hannonlab.cshl.edu/fastx_toolkit/). Both sequence files of each sample were joined and processed on QIIME2 2025.7 amplicon pipeline^46^, removing chimeric sequences and building ASVs (Amplicon Sequence Variant) using the Deblur algorithm. Then, all ASVs were classified using the SILVA v138.2 16S^47^database assuming 97% identity. Before usage, the database was filtered to remove sequences that could be associated with fungi, filtering out Eukaryota, plants, removing Mitochondria and Chloroplast, and sequences with unspecified species classification. After classification, a phylogenetic tree was built using MAFFT (Multiple Alignment using Fast Fourier Transform) and fasttree methods. Sequences that failed to match the database were discarded.

### Data collection and definitions

Clinical data and blood tests at enrolment were captured on standard case record forms. We performed extensive searches of patient contacts and electronic health care databases to ascertain vital status at 12 weeks if a patient was not contactable at 4 and/or 12 weeks. We used a three-point dissemination score previously described^48^ to indirectly quantify the degree of mycobacterial dissemination. The score was calculated by allocating one point for each positive test from the urine Xpert MTB/RIF assay, urine LAM test and mycobacterial blood culture, yielding a score ranging from 0 to 3.

### Statistical Analysis

Comparisons were made between patients hospitalized with a new diagnosis of HIV-TB who died within 12 weeks and survivors and between inpatients and HIV-positive outpatient controls without active tuberculosis. Analyses were performed using R statistical software, Graphpad Prism and JMP Statistical Software (developed by SAS Institute). Non-parametric tests (Kruskal Wallis with Tukey Kramer post hoc test or Wilcoxon rank sum test where appropriate) were used for comparisons. Median values with interquartile range were used as measures of central tendency and number and percentage for categorical variables. Markers of microbial translocation and gastro-intestinal epithelial damage were explored by correlation with baseline clinical characteristics or baseline laboratory measures using Spearman’s rank correlation and were assessed for association with mortality using logistic regression analyses. Multivariable analyses included correction for sex, age, HIV viral load, creatinine and soluble mediators of inflammation (summarised as principal components scores)^4^.

Concentrations of microbial translocation and gastrointestinal epithelial damage markers were plotted against the TB dissemination score. Participants who did not have all three tests to calculate the dissemination score performed were reviewed and it was found that in patients who did not have samples collected for all three tests these samples could not be assumed to be missing at random. We therefore used multiple imputations using Multivariate Imputation by Chained Equations (v3.16.0) in R to impute missing values for the three tests to calculate a dissemination score in all patients. The Jonckheere-Terpstra test for trend was used for comparison of medians across dissemination score groups used as an ordinal categorical variable.

To evaluate differences in blood microbiome biodiversity between the groups alpha-diversity rarefaction curves, Shannon and Inverse Simpson indices as well as Faith’s Phylogenetic Diversity were applied. The sample-size based rarefaction and extrapolation (R/E) sampling curve for amplicon sequence variants (ASVs) richness, based on the Hill number^49^, was generated by the number of observed individuals with iNEXT package in R (v3.6.1). However, the rarefaction curve does not account for the composition of each community, which leads to a skewed estimation of bacterial composition. Thus, the Shannon and Inverse Simpson indices as well as Faith’s Phylogenetic Diversity (to confirm the abundance found by rarefaction curves) were estimated with the microbiome package in R (v3.6.1), then plotted as boxplots and compared using Mann-Whitney in R (v3.6.1). To evaluate whether taxa were capable of differentiating the groups, beta-diversity principal coordinate analysis (PCoA) was applied. Accordingly, Phylogenetic Isometric Log-Ratio (PhILR)^50^ transformation followed by Euclidian distance was measured for all samples and these values were used to create the PCoA plots with Cailliez correction. To determine whether any groups of samples contained significantly different bacterial communities, Permutational Multivariate Analysis of Variance (PERMANOVA) from vegan (v2.7–3; Oksanen et al 2026) package was used. Benjamini-Hochberg correction for multiple testing has been applied to remove false positive differences followed by the homogeneity assessment of group dispersions which was done using Permutational Analysis of Multivariate Dispersions (PERMDISP). To identify the differentially abundant genera between the groups we performed the Negative Binomial GLM fitting, and the log fold-change was computed between the groups using the DESeq2^51^ package in R (v3.6.1). The genus was considered differentially abundant based on |logFC| > 1.4 and the False discovery rate (FDR) value < 0.05 thresholds. The soluble mediator of inflammation concentrations were used as input to calculate the molecular degree of perturbation (MDP) score^52^, using the mdp package in R (v3.6.1), to assess the variation across groups. The MDP score of each individual biomarker was defined by z-score normalization, where the differences in concentration from the average concentration of the biomarker in the reference group (outpatient controls) were divided by the reference standard deviation. The MDP score represents the differences by the number of standard deviations from the outpatient control group.

### Ethical approval

The study was approved by the University of Cape Town Human Research Ethics Committee (UCT HREC reference: 057/2013). Informed consent was taken from all outpatients and all eligible hospitalized patients who were able to provide consent. Eligible hospitalized patients with a decreased level of consciousness were enrolled without consent and followed up daily until they regained capacity to participate in the informed consent process; this was approved by UCT HREC. Permission was obtained from the UCT HREC to use information of participants who died prior to providing informed consent as per the approved protocol.

## Results

**Figure 1:**
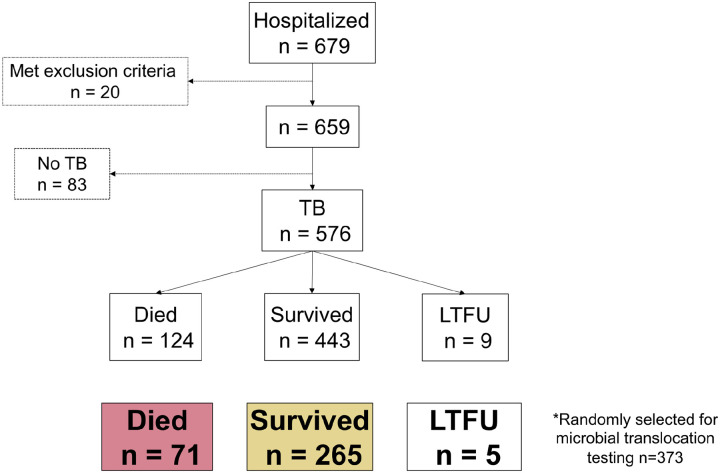
Study Flow chart Patients were enrolled in a prospective observational cohort study investigating the causes of mortality in HIV-positive patients admitted to hospital with HIV-associated tuberculosis. A cohort of n=373 patients were randomly selected and markers of microbial translocation and gastro-intestinal endothelial damage concentrations were measured.

The parent study enrolled n=576 hospitalized with HIV-TB and n=35 outpatient controls with HIV without tuberculosis. Markers of microbial translocation and gastrointestinal epithelial damage were measured in n=373 randomly selected patients with HIV-TB, including n=71 hospitalized patients who died within 12 weeks, n=265 hospitalized patients who survived and n=32 outpatient controls (Figure 1). Baseline clinical characteristics and concentrations of microbial translocation and gastrointestinal epithelial damage markers by group are shown in Table 1. Bacterial 16s ribosomal DNA was measured in n=235 patients which included all patients who died and had stored sample and a random selection of survivors and outpatient controls (n=111 who died, n= 104 survivors and n=20 outpatient controls).

### Markers of microbial product translocation and GIT epithelial damage

**Figure 2:**
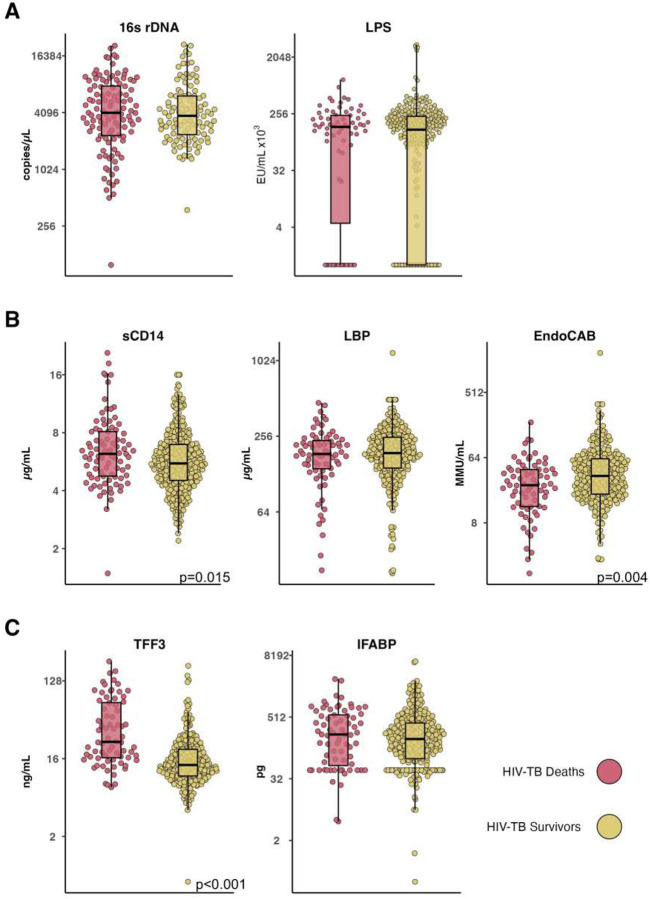
Markers of microbial translocation and gastro-intestinal mucosal damage in HIV-positive patients hospitalized with HIV-associated tuberculosis comparing those who died to those who survived by 12 weeks:

Bacterial 16s rDNA concentrations were significantly higher in hospitalized HIV-TB patients compared to outpatient controls (p=0.003) and there was no significant difference between hospitalized patients who died and those who survived (p=0.655). LPS concentrations were low overall and not significantly different between groups (Table 1 and Figure 2A). Soluble CD14 and LBP concentrations were significantly higher in hospitalized HIV-TB patients compared to outpatients (p<0.001) and sCD14 concentrations were also higher in hospitalized patients who died compared to survivors (p=0.015). EndoCAB concentrations were significantly lower in hospitalized HIV-TB patients compared to outpatients (p<0.001) (Figure 2B) and significantly lower in hospitalized patients who died compared to survivors (p<0.004). TFF3 concentrations were significantly higher in hospitalized HIV-TB patients who died compared survivors (p <0.001) and significantly higher in hospitalized patients compared to outpatient controls (p<0.001). IFABP was unexpectedly significantly higher in outpatient controls compared to hospitalized patients (p <0.001), but was not different between those who died and survived (Figure 2C).

### Correlation and logistic regression analyses

Correlation analyses were performed between full blood count, renal function, liver function, soluble markers of inflammation and markers of microbial translocation and gastro-intestinal epithelial damage. Trefoil factor 3 showed a strong positive correlation with urea and creatinine (Spearman’s rho = 0.61 and 0.60 respectively) and 16s rDNA concentrations showed strong positive correlation with white cell count and neutrophil count (Spearman’s rho = 0.88 and 0.87 respectively).

Univariable logistic regression analysis showed significantly increased odds of mortality per log2 sCD14 pg/mL increase in concentration (Odds ratio [OR]= 1.8, 95% confidence interval [CI]: 1.16 – 2.78, Wald’s p=0.009), per log2 TFF3 pg/mL increase in concentration (OR = 2.56, 95% CI: 1.96 – 3.36, p < 0.001) and significantly decreased odds of mortality per unit increase in log2 EndoCAB MMU/mL concentrations (OR = 0.72, 95% CI: 0.58, 0.88, p=0.002). In multivariable logistic regression analyses adjusted for age, sex, HIV viral load, creatinine and soluble mediators of inflammation concentrations, the association of sCD14 and EndoCAB with mortality was abrogated. TFF3 remained independently associated with mortality when adjusted for these clinical variables (Table 2).

### Metagenomic sequencing

**Figure 3:**
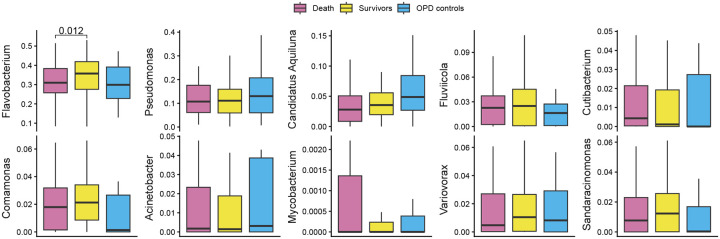
Bacterial DNA: Top ten relative abundant genus in HIV-positive patients hospitalized with HIV-associated tuberculosis and HIV-positive outpatients without tuberculosis: Box plots from the top ten most abundant bacteria across the hospitalized patients who died within 12 weeks (Death), those who survived 12 weeks (Survivors) and the HIV-positive outpatient controls without tuberculosis (OPD controls). The comparisons were performed using Kruskall-Wallis test and Tukey-Kramer post-hoc. The significant p-values were plotted in the graphs.

Comparing the 10 most abundant bacterial genera amongst hospitalized HIV-TB patients, *Flavobacterium* was significantly lower in hospitalised patients with HIV-TB who died within 12 weeks compared to survivors (Figure 3).

### Bacterial diversity

**Figure 4:**
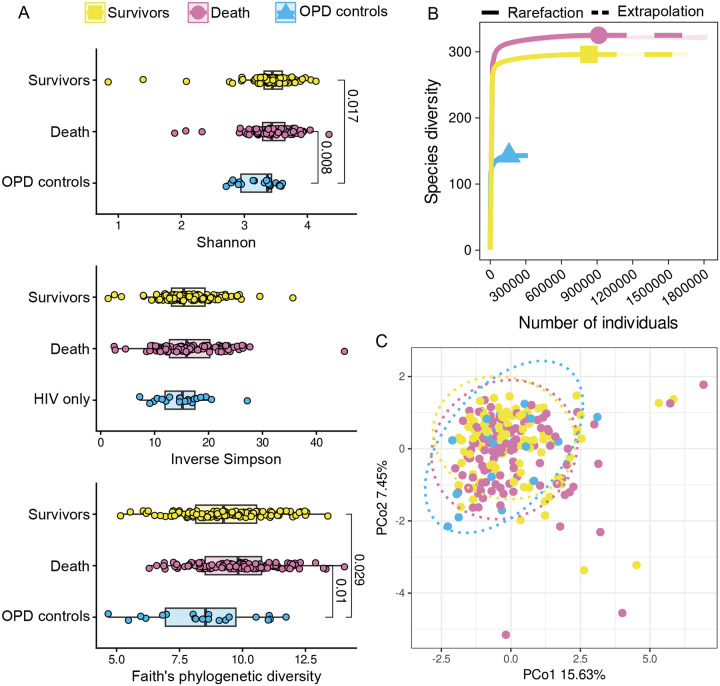
Bacterial DNA: Alpha and Beta Diversity Bacterial diversity analysis from the hospitalized HIV-TB patients who died within 12 weeks (Death), those who survived 12 weeks (Survivors) and the HIV-positive patients with tuberculosis outpatient controls (OPD controls): Panel A: violin plots displaying the Shannon, Inverse Simpson and Faith’s phylogenetic diversity alpha diversities metrics. The comparisons were performed using Kruskal-Wallis test and Tukey-Kramer post-hoc. The significant p-values are shown in graphs. Panel B: The number of operational taxonomic units as a function of the number of samples rarefaction curve. Panel C: Beta diversity principal coordinate analysis (PCoA) plots were calculated using the weighted UniFrac distance.

The difference between the bacterial diversity across the groups (alpha diversity) was estimated by the Shannon, Inverse Simpson and Faith’s phylogenetic diversity indexes (Figure 4A). The hospitalized patients with HIV-TB who died within 12 weeks showed significantly higher diversity compared to HIV positive controls in the Shannon and Faith’s phylogenetic indexes. Hospitalised HIV-TB patients had significantly higher diversity compared to outpatient control patients. The operational taxonomic unit’s richness curve based on the Hill number was applied (Figure 4B). The rarefaction curve showed that hospitalized HIV-TB patients who died had higher diversity, followed by HIV-TB survivors and the lowest diversity was seen in the HIV-positive controls. The beta-diversity analysis was applied to verify the compositional heterogeneity of bacteria communities amongst the groups. Principal coordinates analysis using weighted Unifrac distances was performed (Figure 4C) and did not show a shift between groups superior to the interindividual variability.

**Figure 5:**
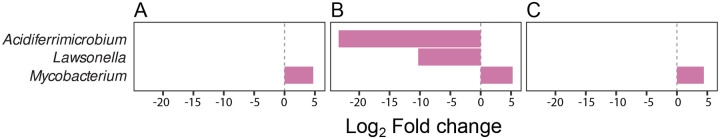
Differential abundance of bacteria in patients hospitalized with HIV-TB who died (all, early, and late deaths) within 12 weeks and those who survived. Differential abundance analysis: A - Bar plots from the bacterial composition log2 fold change variation in hospitalized HIV-TB patients who died within 12 weeks compared to survivors. B - Bar plots from the bacterial composition log2 fold change variation in early deaths compared to survivors. C - Bar plots from the bacterial composition log2 fold change variation in late deaths compared to survivors The differentially abundant taxa were identified though analysis of DESeq2. All genera with statistically significant p-values and ≥ 1.4 and ≤ −1.4 fold change are shown in graphs. Positive log2FC indicate genera which were less abundant in hospitalized HIV-TB patients who died within 12 weeks and negative log2FC indicate genera which were more abundant in patients who died within 12 weeks.

We assessed the differential abundance of bacteria between patients hospitalized with HIV-TB who died within 12 weeks compared to patients who survived 12 weeks. *Mycobacterium genus*, which includes *Mycobacterium tuberculosis* species, was significantly more abundant in patients who died (Figure 5A). Stratified analysis also demonstrated higher relative abundance in both early and late death groups (Figures 5B and 5C).

### Microbial translocation markers and markers of tuberculosis dissemination

**Figure 6:**
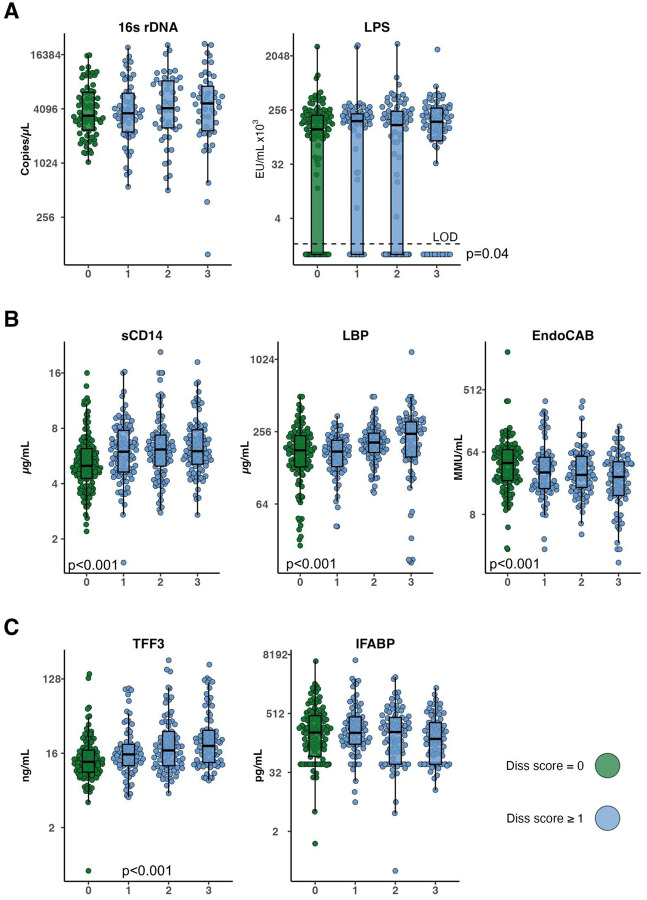
TB Dissemination score and markers of microbial translocation and gastro-intestinal mucosal damage in HIV-positive patients hospitalized with HIV-associated tuberculosis: Boxplots with overlaid violin plots show comparisons of microbial translocation marker concentrations amongst patients with tuberculosis dissemination scores ranging from 0 to 3. The 3 point tuberculosis dissemination score was calculated by allocating one point for each positive test amongst mycobacterial blood culture, urine lipoarabinomannan (LAM) and urine Xpert tests. Jonckheere-Terpstra test for trend across ordinal categorical variables was performed and p-value displayed if significant. **Panel A** shows 16s rDNA (bacterial 16s ribosomal DNA concentrations) which was measured in a sub-group of n= 235 patients and LPS (lipopolysaccharide) concentrations. **Panel B** shows concentrations of indirect markers of microbial translocation: sCD14: soluble CD14; LBP: lipopolysaccharide binding protein and EndoCAB: endotoxin core antibody IgM. **Panel C** shows markers of gastro-intestinal mucosal damage: TFF3: trefoil factor 3 and IFABP: intestinal fatty acid binding protein. Copies/μL: copies per microliter of whole blood; EU/mL: Endotoxin units per millilitre; μg/mL: microgram per millilitre; MMU/mL: standard median units per millilitre; ng/mL: nanogram per millilitre; pg/mL: picogram per millilitre.

**Figure 7:**
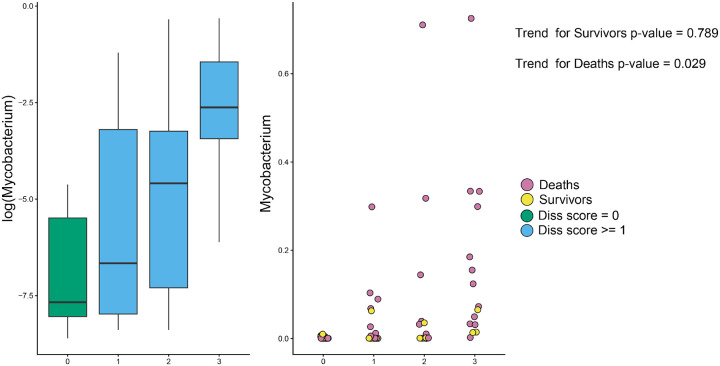
Mycobacterium relative abundance vs dissemination score Boxplot of *Mycobacterium* abundance and the tuberculosis dissemination score with tuberculosis dissemination scores ranging from 0 to 3. The second panel shows the same comparison coloured by outcome with deaths in red and survivors in yellow. The 3 point tuberculosis dissemination score was calculated by allocating one point for each positive test amongst mycobacterial blood culture, urine lipoarabinomannan (LAM) and urine Xpert tests Jonckheere-Terpstra test for trend across ordinal categorical variables was performed for deaths and survivors.

We plotted all markers against the tuberculosis dissemination score. LPS, soluble CD14, LBP and TFF3 concentrations showed a significant upward trend across groups of patients with dissemination score ranging from 0 to 3. EndoCAB concentrations showed a significant downward trend across groups (Figure 6). Comparing the *Mycobacterium* relative abundance across groups with different tuberculosis dissemination scores, as would be expected, there was a significant upward trend of *Mycobacterium* abundance amongst patients who died within 12 weeks. This trend was mainly driven by higher *Mycobacterium* abundance in the patients who tested positive for all three markers of disseminated tuberculosis. (Figure 7).

### Molecular degree of perturbation

The concentrations of soluble mediators of inflammation were used to calculate a molecular degree of perturbation (MDP) score to quantify the heterogeneity in samples. There was a stepwise increase in the degree of perturbations from outpatient controls to hospitalized survivors and hospitalized deaths (Supplementary Figure 4). Hospitalized patients who died had significantly more heterogeneous results compared than hospitalized patients who survived 12 weeks.

**Figure 8:**
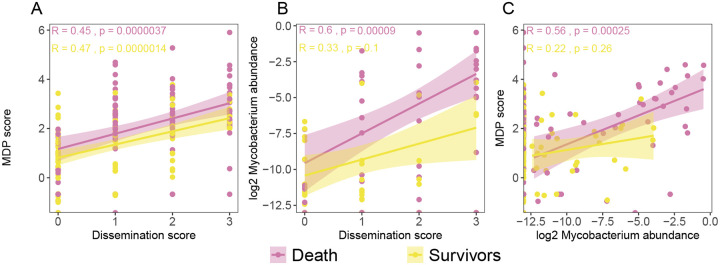
MDP score association with Dissemination score and Mycobacterium 16s rDNA abundance in blood **Associations between immune perturbation, disease dissemination, and Mycobacterium abundance in HIV-TB patients stratified by outcome:** (A) Scatterplots showing the correlation between the molecular degree of perturbation (MDP) of immunological markers and the dissemination score in HIV-TB patients who survived and those who died.(B) Correlation between the relative abundance of Mycobacterium and the dissemination score stratified by outcome.(C) Correlation between the relative abundance of Mycobacterium and the MDP score of immunological markers. Lines represent linear regression fits with 95% confidence intervals. Correlation coefficients and corresponding p values were calculated using Spearman’s rank correlation test. Each point represents an individual participant.

Immune perturbation, quantified using MDP score of immunological markers, was significantly associated with the dissemination score in patients with HIV-TB in both outcome groups (Figure 8A). A positive correlation between MDP and dissemination score was observed among patients who survived as well as among those who died, indicating that increasing systemic immune perturbation is linked to greater disease dissemination regardless of clinical outcome.

In contrast, the relationship between microbial DNA burden and disease dissemination differed by outcome. The relative abundance of *Mycobacterium* was significantly correlated with the dissemination score only among patients who died, whereas no significant association was observed in patients who survived (Figure 8B). This suggests that higher blood mycobacterial DNA abundance is more closely linked to systemic dissemination in fatal cases.

Consistent with this pattern, the relative abundance of *Mycobacterium* was also positively correlated with the MDP score of immunological markers exclusively in patients who died, while no significant association was detected among survivors (Figure 8C).

## Discussion

We analysed markers of gastro-intestinal epithelial damage, microbial translocation and the blood bacterial microbiome (measuring DNA) in patients hospitalised with HIV-associated tuberculosis and compared hospitalised patients who died within 12 weeks to survivors and to outpatient controls without TB. Soluble CD14 was significantly higher and EndoCAB significantly lower in patients who died compared to survivors and TFF3, a marker of gastro-intestinal epithelial damage, was independently associated with mortality. LPS, sCD14, LBP, and TFF3 showed a significant upward trend and EndoCAB a significant downward trend when plotted against the tuberculosis dissemination score. Hospitalised patients who died had higher bacterial diversity but the bacterial communities were homogeneous between the outcome groups. *Mycobacteria* were the only differentially more abundant bacteria in hospitalised patients who died and was significantly higher in patients with higher dissemination scores. Hospitalised patients who died had the highest immune perturbation score.

Using soluble mediators of inflammation values and a molecular degree of perturbation score, we found the highest degree of heterogeneity in the samples of patients who died. Moreover, this systemic immune perturbation was closely associated with the extent of TB dissemination, independent of clinical outcome. Increased immune activation is a hallmark of HIV disease and is further amplified with TB, with several studies demonstrating that elevated inflammatory and immune activation markers correlate with disease severity and mortality risk in HIV-associated TB^53,54^. In this context, the positive association observed between the molecular degree of perturbation (MDP) of immunological markers and the dissemination score in both survivors and patients who died suggests that systemic immune disturbance reflects the overall burden of disseminated disease. Indeed, systemic inflammatory signatures have been associated with more severe TB presentations and adverse outcomes^52,55^. However, the relationship between microbial DNA load in blood and disease dissemination differed according to outcome: the relative abundance of *Mycobacterium* correlated with dissemination exclusively among patients who died. HIV infection is known to impair key mechanisms required for containment of *M. tuberculosis*, including macrophage function and T-cell responses, thereby facilitating bacterial replication and systemic spread^54,56^.

Consistent with this pattern, the abundance of *Mycobacterium* was also associated with immune perturbation only among patients with fatal outcomes, suggesting a convergence between microbial burden and systemic immune dysregulation in more severe disease. Previous work has shown that TB in people living with HIV can drive sustained inflammatory activation that contributes to immunopathogenesis and poor clinical outcomes^57^. Moreover, dysregulated inflammatory networks and immune activation profiles have been linked to disease severity and mortality in TB^55^. The coupling observed here between mycobacterial abundance, immune perturbation, and dissemination in patients who died may therefore reflect a pathological feedback loop in which increasing pathogen burden amplifies systemic inflammation while the impaired immune response fails to effectively control infection. In contrast, the absence of this association in survivors may indicate partial preservation of host–pathogen homeostasis, in which immune perturbation occurs but is less coupled with bacterial burden and widespread dissemination.

We found no association of 16s rDNA concentrations with mortality or with biomarkers of tuberculosis dissemination. Bacterial 16s rDNA can be measured in whole blood, is present in healthy people^41^ and has been linked to diseases in which microbial translocation plays a role in pathogenesis, including liver disease, cardiometabolic disease, chronic kidney failure, depression, cancer and COVID-19^43,58–64^. Studies which report 16s rDNA concentrations in HIV-positive patients have not been done in similar settings to our study which included patients hospitalised with advanced HIV and tuberculosis. The median 3736.7 copies/μL of whole blood in our study is far higher than 16s rDNA concentrations found in the plasma of HIV-positive patients in a US study (median 132 copies/μL of plasma)^8^ and higher than in HIV-hepatitis C co-infected patients in a Spanish study (median 198.87 copies/μL of plasma^65^). However, direct comparison of results from whole blood and plasma may be inappropriate due to technical differences of the PCR assays, standard curves and normalization of the values. Our findings are similar to findings from the SMART-trial which showed no association of 16s rDNA concentrations in plasma with poor clinical outcome^9^. The lack of association with biomarkers of tuberculosis dissemination, which include mycobacterial blood culture positivity, is likely explained by *Mycobacterial tuberculosis* DNA making a relatively small contribution to total blood 16s rDNA concentration even when there is mycobacteraemia^41^. In the parent cohort 88% of patients received ceftriaxone during the index admission, often before study enrolment, and this may have affected the bacterial 16s rDNA concentration findings. The role of 16s rDNA in identification of pathogenic bacterial bloodstream infections has been previously studied with variable findings^66–69^.

We found low concentrations of lipopolysaccharide (LPS) across this cohort with no differences between outcome groups, however there was a significant upward trend in LPS concentrations as number of positive biomarkers of tuberculosis dissemination increased. LPS is a potent stimulator of the immune system and is rapidly bound by antibodies and binding proteins. Depending on the concentration, relative binding and clearance from the circulation, LPS variably triggers pro-inflammatory responses. To trigger an immune response LPS is recognized by sCD14 or LBP and transferred to membrane bound CD14 which acts as an LPS receptor on myeloid cells. Membrane bound CD14 binds LPS to the TLR4/MD2 complex, which activates nuclear factor-κB (NF-κB) through a myeloid differentiation primary response gene 88 (MyD88) dependent or MyD88-independent pathway to mediate secretion of pro-inflammatory mediators such as interleukin-1β (IL-1β), IL-6, TNF-α and type 1 interferon^70^. To clear LPS from circulation, sCD14 or LBP transfer LPS to high density (HDL) or other lipoproteins in plasma and these bind LPS in such a way that it cannot interact with the TLR4/MD2 complex^40^. All LPS assays, including the LAL assay we used, are technically challenging, sensitive to interference and therefore have caveats in interpretation. The LPS molecules produced by bacteria, the LPS used to prepare assay standards and the LPS measured in plasma are heterogenous molecules. LPS consists of a lipid A, core and O-antigen, but LPS molecules (even LPS extracted from pure cultures) have variation in the polysaccharide chain length, acetylation of lipid A and the extent to which molecules such as phosphate, ethanolamine and arabinose are attached to the lipid A or core oligosaccharide^40^. Some LPS molecules may stimulate (or activate blood cells), some LPS molecules do not stimulate blood cells and some may even be inhibitory. None of the LPS assays can distinguish these types of LPS molecules and this limits the interpretation of LPS measurements^40,71^. LPS concentrations are elevated in HIV infection and previous cohort studies which measured LPS concentrations in HIV-TB patients showed high LPS concentrations, thus we expected to find high concentrations of LPS in this cohort of acutely ill patients hospitalized with HIV-TB^6,72,73^ but instead found very low concentrations. Technical issues with performance of the assay cannot be ruled out. It is also possible that a constituent of plasma present in high abundance in our patient population which was not neutralized by heat inactivation interfered with the activation of LAL and detection of LPS in our samples. On the other hand, these findings may accurately reflect low concentrations of LPS due to successful clearance from the peripheral blood by sCD14 and LBP, both of which we found in high concentrations and both of which have been shown to remove LPS from cell surfaces and to reduce the bioactivity of LPS^35,74–77^. High concentrations of LBP have also been shown to prevent LPS from activating LAL^78^. The upward trend in patients with increasing numbers of positive markers for dissemination may be due to more severe gastro-intestinal epithelial damage as reflected by the higher concentrations of TFF3 across the same groups.

We found high concentrations of sCD14 and LBP in hospitalized HIV-TB patients compared to outpatient controls and sCD14 concentrations were higher in patients who died compared to survivors. Both markers showed a significant upward trend in concentrations with increasing numbers of positive biomarkers of disseminated tuberculosis. Soluble CD14 is produced by activated monocytes and macrophages and is a non-specific marker of monocyte/macrophage activation^79^. Higher sCD14 concentrations are associated with disease progression and poor outcome in patients with chronic HIV infection^9,80^ but this is unlikely a causal relationship. Higher concentrations of sCD14 are taken to indicate a state of chronic immune activation and microbial translocation is one of the underlying causes of immune activation in HIV. Soluble CD14 concentration is also elevated in active tuberculosis infection^81^. LBP is an acute phase protein which is produced by the liver as part of the immune response to gram-negative bacterial infection^82^. Both sCD14 and LBP have the ability to stimulate or inhibit the immune response to LPS and the nature of the response depends on the concentration of both markers and their environment^75^. High concentrations of LBP and sCD14 during acute bacterial infection inhibit the systemic cellular immune response to LPS^83 84^, whereas low concentrations at the site of infection or in tissue may enhance the LPS-associated immune activation of monocytes and macrophages^36,75^. sCD14 can remove LPS from cellular CD14 and transfer LPS to plasma lipoproteins^35^. Both sCD14 and LBP concentrations are used as surrogate markers of microbial translocation in the peripheral blood, however both markers are non-specific and could be increased due to the acute phase response to other infections such as bacterial infections or TB. Our findings of low LPS concentration and high concentrations of LBP and sCD14 are similar to a study in Uganda which also found unexpectedly low LPS concentrations in patients with HIV-TB compared to patients with only HIV infection and a CD4 count <350 cells/μL. They found higher concentrations of LBP in HIV-TB, but did not find a negative correlation between plasma LBP concentration and LPS concentration, so postulated that other LPS binding proteins, in addition to LBP, were implicated in their finding of lower than expected LPS concentrations^73^.

We found significantly lower endotoxin core antibody (EndoCAB) IgM concentrations in hospitalized HIV-TB patients who died and a significant trend towards lower concentrations in patients with increasing numbers of positive biomarkers of disseminated tuberculosis. EndoCAB is involved in clearing LPS from systemic circulation and EndoCAB IgA, IgM and IgG can be measured in healthy individuals^85^. Several studies show a drop in EndoCAB levels after major surgery and this is thought to be due to EndoCAB consumption by binding to LPS that is released into the circulation during the postoperative period^86^. EndoCAB which is bound to LPS is not measured by the EndoCAB assay. Studies have also shown that people with low pre-operative EndoCAB values and sepsis patients with low EndoCAB concentrations have poor clinical outcomes compared to people with normal values^87–89^. This has been interpreted as an indication that patients with low EndoCAB concentrations do not have the same capacity or reserves to deal with the post-operative or sepsis related systemic release of LPS. This finding supports increased microbial translocation in hospitalized patients who died and in patients with disseminated tuberculosis, despite our not finding elevated concentrations of LPS in these patient groups.

We found higher concentrations of Trefoil factor 3 (TFF3) in hospitalized HIV-TB patients who died compared to those who survived, an independent association with 12-week mortality and a significant upward trend in patients with more positive biomarkers of disseminated tuberculosis. Trefoil factor 3 (TFF3) is a stable secretory protein which is produced by gastrointestinal mucosal cells. It is part of the trefoil family of proteins which are secreted by mucin-producing gastrointestinal cells and play a key role in the maintenance and repair of the gastrointestinal mucosa^37^. TFF3 expression increases when gastrointestinal mucosal damage occurs and is used as a marker of gastrointestinal mucosal damage, however TFF3 is also increased in chronic kidney disease, carcinomas and may play a role in oncogenesis of gastric cancers^90–92^.

Intestinal fatty acid binding (IFABP) is a commonly studied marker of gastrointestinal mucosal damage in the context of HIV infection and microbial translocation. Our finding of significantly higher concentrations of IFABP in outpatient controls was unexpected. Fatty acid binding proteins are low molecular weight intracellular proteins which play a role in the metabolism and transport of long chain fatty acids and are tissue specific. IFABP is present in the epithelial cells (enterocytes) of the gastrointestinal system and is rapidly released into systemic circulation when enterocyte necrosis occurs^38^. We found IFABP ~200 pg/mL in hospitalized patients and ~ 600 pg/mL in outpatient controls. Another South African cohort of hospitalized HIV-TB patients found median IFABP concentrations of 137 pg/mL in patients who died and 0 pg/mL in survivors^72^. A cohort study of HIV-TB outpatients in Botswana reported a median concentration of 345 pg/mL in ART naïve patients and 612 pg/mL in a sub-group of patients at 4 weeks on ART^93^. This variability suggests that there may be other biological mechanisms or technical factors that determine measured IFABP concentrations and further research is needed to assess the utility and interpretation of IFABP measurements in the context of HIV, HIV-TB and other co-infections. A plausible hypothesis is that the lower concentrations among inpatients with HIV-TB represents substantial gut atrophy and loss of enterocytes (the cellular source of IFABP) during the illness that leads up to admission, such that by the time of admission IFABP concentrations are lower than HIV-positive outpatient controls.

Although overall bacterial community structure was comparable between groups, *Mycobacterium* was the only taxon that showed a statistically significant increase in abundance among hospitalized patients who died. The parent study investigated causes of mortality in hospitalised patients with HIV-TB and did not find that co-infections such as cryptococcosis, cytomegalovirus or bacterial infections played a major role in mortality. The findings from this study further supports the conclusion from that study that tuberculosis itself is the major cause of mortality in these patients.

Strengths of this study are that it was a large cohort of HIV-positive patients enrolled early after presentation to hospital with extensive tuberculosis diagnostic work up and microbial translocation was assessed by testing seven different markers. Vital status at 12 weeks was known for most of the cohort. Limitations of the study are that no longitudinal sampling was performed, and assessment of microbial translocation markers were not performed on the entire cohort, however measures to minimize selection bias were implemented.

Our findings of higher concentration of TFF3 and lower concentration of EndoCAB support the hypothesis that there is gastrointestinal mucosal damage and increased microbial translocation in patients hospitalized with HIV-TB who died within 12 weeks compared to those who survived. TFF3 was independently associated with mortality which suggests that gastrointestinal epithelial damage may play a role in mortality. Our findings of a significant trend to higher concentrations of LPS and TFF, and lower concentrations of EndoCAB in patients with higher numbers of positive markers of disseminated tuberculosis, support the hypothesis that microbial translocation and gastrointestinal mucosal damage is more severe in patients with a higher systemic mycobacterial load. The finding of differentially more abundant *Mycobacterium* in hospitalised patients who died support the hypothesis that tuberculosis is the predominant cause of death in these patients. Mycobacterial relative abundance and the immune perturbation score increased with increased numbers of dissemination markers which indicates a link between a higher mycobacterial load and more severe immune dysregulation. An improved understanding of the pathophysiology related to mortality in hospitalized patients with HIV-TB is a research priority and urgently needed to direct the development of improved treatment strategies in this patient population.

## Supplementary Material

Supplementary Files

This is a list of supplementary files associated with this preprint. Click to download.

- 2.MTLpaperSupplementaryFigures2May2026.pdf
- 4.DataFig34578Supp2data.xlsx
- 5.DataFig1267.xlsx

## Figures and Tables

**Table 1:** Clinical characteristics and concentrations of microbial translocation and gastrointestinal epithelial damage markers

	Hospitalized HIV with Active TB Died n = 71	Hospitalized HIV with Active TB Survived n = 265	Outpatient HIV without TB n = 32	p^a^ Died vs Survived	p^b^ Hosp vs OPD
**Sex, Female**	37 (52.1)	130 (49.1)	23 (71.9)	0.690	**0.025**
**Age, years**	41 [35, 47]	35 [31, 43]	35 [30, 42]	**<0.001**	0.680
**CD4 count, cells/μL**	35 [17, 77]	67 [24, 133]	168 [102, 229]	**<0.001**	**<0.001**
**HIV viral load, log**_**10**_ **copies/mL**	4.98 [3.29, 5.74]	5.11 [3.43, 5.75]	4.27 [1.59, 4.82]	0.567	-
**C-reactive protein, mg/L**	175 [109, 249]	145 [90, 211]	-	**0.008**	-
**Venous lactate, mmol/L**	2.40 [1.55, 3.45]	1.70 [1.30, 2.40]	-	**<0.001**	-
**Creatinine, mmol/L**	108 [68, 183]	76 [59, 107]	-	**<0.001**	-
**Haemoglobin, g/dL**	7.80 [6.70, 10.05]	9.00 [7.40, 10.60]	12.10 [10.80, 13.27]	**0.016**	**<0.001**
^c^ **Whole blood bacterial 16s rDNA, copies/μL**	3962.71 [2407.90, 7640.73]	3877.16 [2338.93, 6167.42]	2195.98 [1456.43, 3466.72]	0.655	**0.002**
**LPS, EU/mL**	0.16 [0.01, 0.24]	0.14 [0.00, 0.23]	0.11 [0.00, 0.16]	0.512	0.086
**sCD14, μg/mL**	6.21 [4.76, 8.11]	5.54 [4.52, 6.95]	3.66 [2.59, 4.39]	0.015	**<0.001**
**LBP, μg/mL**	185.02 [141.20, 236.66]	188.11 [142.97, 251.19]	103.23 [84.07, 133.07]	0.634	**<0.001**
**EndoCAB, MMU/L**	26.60 [13.50, 43.80]	35.70 [20.00, 61.70]	100.35 [62.73, 208.45]	**<0.004**	**<0.001**
**TFF3, ng/mL**	25.06 [16.40, 71.73]	13.52 [10.09, 20.54]	10.97 [9.71, 13.22]	**<0.001**	**<0.001**
**IFABP, pg/mL**	234.12 [58.21, 564.89]	190.85 [78.92, 392.10]	610.76 [385.69, 937.29]	0.362	**<0.001**

Five inpatient participants were lost to follow-up and are not included in this table.

Categorical variables are presented as count and percentage, n (%). Continuous variables are presented as median with IQR: median [IQR].

a The p-value was calculated by comparing hospitalised HIV-TB patients who died within 12 weeks to patients who survived.

b The p-value was calculated by comparing hospitalised patients HIV-TB patients to outpatient HIV positive patients without TB.

LPS: lipopolysaccharide; EU/mL: Endotoxin units per millilitre; sCD14: soluble CD14; μg/mL: microgram per millilitre; LBP: lipopolysaccharide binding protein; EndoCAB: endotoxin core antibody IgM; MMU/mL: standard median units per millilitre; TFF3: trefoil factor 3; ng/mL: nanogram per millilitre; IFABP: intestinal fatty acid binding protein; pg/mL: picogram per millilitre; Whole blood bacterial 16s rDNA: bacterial 16s ribosomal DNA; copies/μL: copies per microliter of whole blood.

c Bacterial 16s ribosomal DNA was quantitated in n=235 participants which consisted of all who died and had an available sample and a random selection of survivors and outpatient controls (n=111 who died, n= 104 survivors and n=20 outpatient controls).

**Table 2:** Logistic regression analysis of microbial translocation and gastrointestinal epithelial damage markers and 12-week mortality

	Univariable OR (95%CI)	p	Multivariable OR (95%CI)	p
16s rDNA copies/μL	0.97 (0.77, 1.21)	0.767	-	-
LPS EU/mL	0.77 (0.33, 1.82)	0.527	-	-
sCD14 pg/mL	1.80 (1.16, 2.78)	**0.009**	1.01 (0.57, 1.78)	0.963
LBP ng/mL	0.88 (0.64,1.21)	0.435	-	-
EndoCAB MMU/mL	0.72 (0.58, 0.88)	**0.002**	0.92 (0.71,1.19)	0.520
TFF3 pg/mL	2.56 (1.96,3.36)	**<0.001**	1.93 (1.27,2.92)	**0.001**
IFABP pg/mL	1.04 (0.90,1.20)	0.562	-	-

Univariable and multivariable logistic regression analysis results. Each marker with a significant odds ratio in univariable analysis was adjusted for age, sex, HIV viral load, creatinine and principal components 1, 2 and 3 (which encapsulated the variance in 28 soluble mediators of inflammation concentrations) in a multivariable analysis. All microbial translocation variables were log2 transformed for this analysis, except LPS due to the high number of values which equaled zero in the LPS variable.

## Data Availability

Translocation marker data will be made publicly available via UCT’s ZivaHubb platform.
